# Mechanism of action and future perspectives of ADCs in combination with immune checkpoint inhibitors for solid tumors

**DOI:** 10.1007/s10238-025-01655-6

**Published:** 2025-05-04

**Authors:** Yahui Lv, Xiaoran Cui, Tao Li, Chang Liu, An Wang, Ting Wang, Xin Zhou, Ruixin Li, Fan Zhang, Yi Hu, Tong Zhang, Zhefeng Liu

**Affiliations:** 1 Senior Department of Oncology, The First Medical Center, Chinese PLA General Hospital, Beijing, 100853 China; 2 Chinese PLA Key Laboratory of Oncology, Key Laboratory for Tumor Targeting Therapy and Antibody Drugs (Ministry of Education, China), Beijing, China; 3 Medical School of Chinese PLA, Beijing, 100853 China; 4 Senior Department of Oncology, The Fifth Medical Center, Chinese PLA General Hospital, Beijing, 100853 China; 5 Department of Stomatology, The First Medical Center of PLA General Hospital, Beijing, 100853 China; 6 Changchun Veterinary Research Institute, Key Laboratory of Jilin Province for Zoonosis Prevention and Control, Chinese Academy of Agricultural Sciences, Yujinxiang Street 573, ChangchunJilin, 130122 China; 7 Senior Department of Oncology, The Third Medical Center, Chinese PLA General Hospital, Beijing, 100853 China; 8 School of Medicine, Nankai University, TianJin, 30071 China

**Keywords:** Antibody-drug conjugates, Immunotherapy, Immune checkpoint inhibitors, Solid tumor

## Abstract

Antibody–drug conjugates (ADCs) are a promising cancer therapy for targeted delivery of drugs to tumor cells. However, resistance to ADCs remains a challenge, necessitating the exploration of combination therapies. A strong biological theory suggests that ADCs interact with cancer cells and immune cells by triggering mechanisms such as immunogenic cell death, dendritic cell activation, and memory T-cell activation, resulting in long-term anti-tumor immunity and ultimately potential synergistic effects with immunotherapy. Based on extensive and reliable preclinical data, several clinical trials are currently combining ADCs with immune checkpoint inhibitors (ICIs) for the treatment of various cancers, including breast, gastric, and non-small-cell lung cancers, to evaluate the safety and anti-tumor activity of the combination therapy. Preliminary evidence from early clinical trials has reported more effective efficacy data. This paper reviews the combination of ADCs and immunotherapy, highlights the key mechanisms by which the two act synergistically, and summarizes the available clinical evidence against different ADCs targets. The paper also explores the re-challenges used for combination therapies and optimized design options for ADCs drugs.

## Introduction

**Fig.1:**
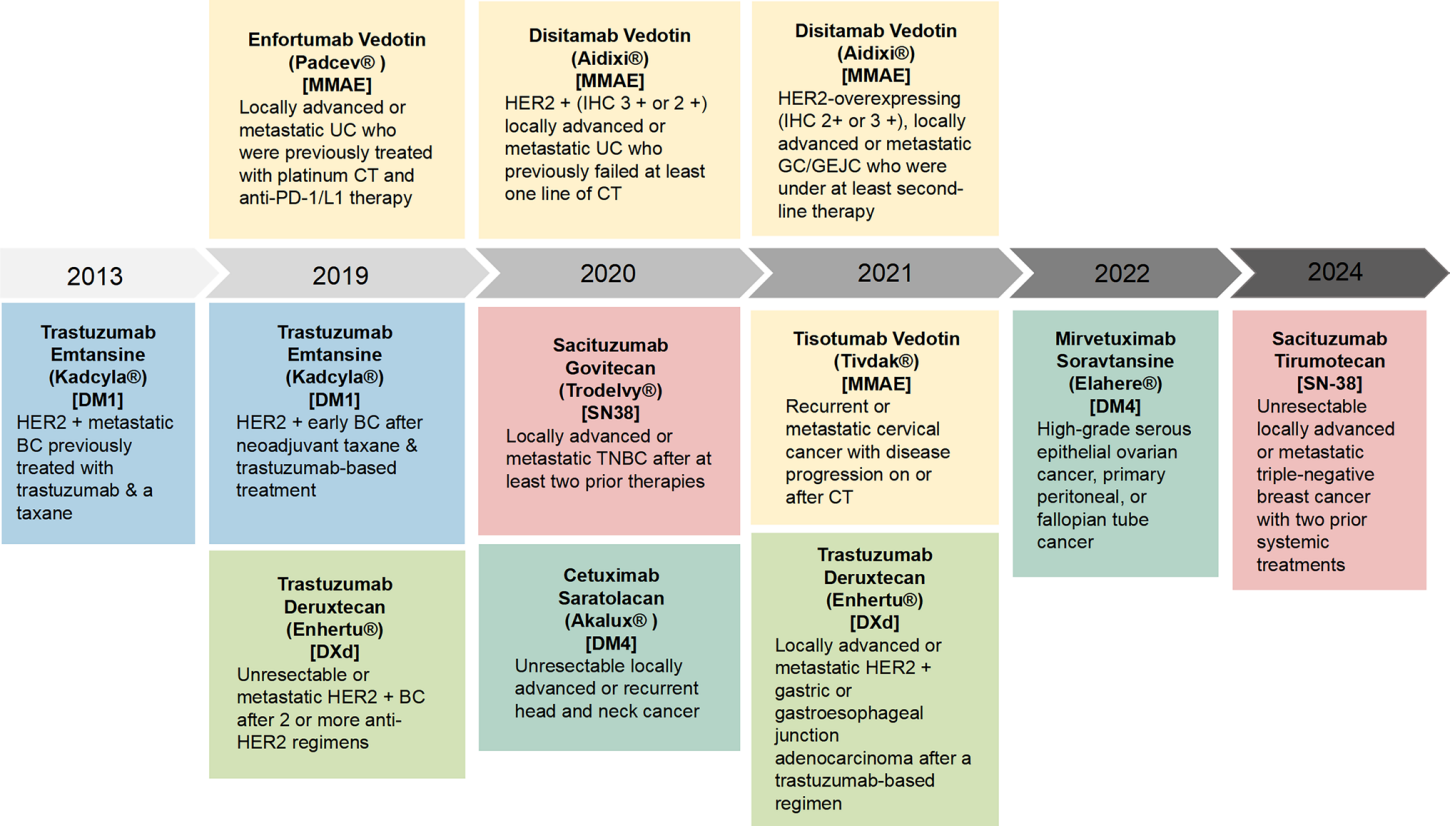
ADC drugs approved for solid tumors

ICIs, represented by PD-1/PD-L1 inhibitors, are a class of drugs that activate immune cells to exert anti-tumor effects by blocking the binding of immune checkpoints to their ligands and relieving immune suppression caused by immune checkpoints [1]. Unfortunately, however, only a fraction of patients could be able to achieve clinical benefit, and there is still a need to explore combination therapy with other drugs or different therapeutic strategies to overcome their resistance and expand their clinical utility [2, 3]. In recent years, novel antibody-based macromolecular complexes represented by ADCs have attracted increasing attention. The drug is composed of an antibody, a payload (a highly potent cytotoxic drug molecule) and a linker connecting the two [4]. The mechanism of action utilizes the binding of antibodies to antigens on the surface of target cells, where ADCs are internalized and release their payloads, thus exerting cytotoxic effects [5], providing an efficient and precise therapeutic strategy for a wide range of solid tumors [6]. Since the first approval of ADCs for the treatment of solid tumors in 2013 [7], nine ADCs have been approved for advanced solid tumors with different indications, which was showed in the Fig. 1. However, drug resistance is still difficult to avoid [8]. Relevant studies have shown that ADCs not only trigger tumor cell death by targeting antigens and releasing payloads, but also induce immunogenic cell death (ICD), which alters the tumor microenvironment and enhances the anti-tumor immune response. ICIs could release immunosuppression and further exert synergistic effects by activating T cells [9, 10], and the combined application of the two has become a research hotspot in the field of cancer therapy. This combination therapy has demonstrated significant efficacy against refractory solid tumors in several clinical trials, along with a favorable safety profile. This review will focus on the research progress of ADCs in combination with immunotherapy, focusing on its mechanism of action, clinical trial results and future development direction, providing new ideas for individualized cancer treatment.

## Biological mechanisms of ADCs combined with ICIs

**Fig. 2:**
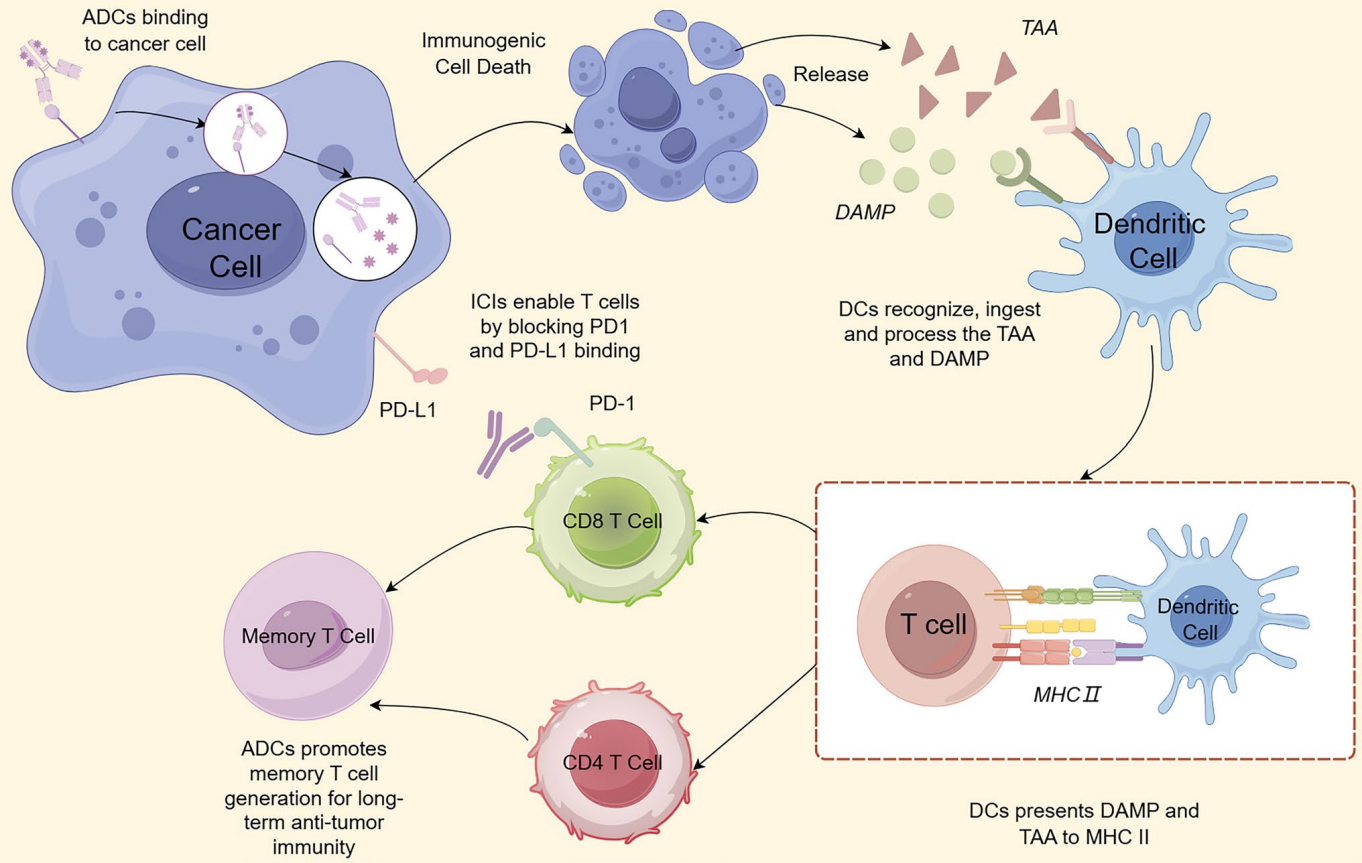
Rationale for combination immunotherapy and antibody–drug concatenates (ADCs)

As shown in the Fig. 2, ADCs are internalized by recognizing and binding to specifc antigens on tumor cells. Subsequently, the linkers of ADCs are degraded, releasing ADCs payloads that trigger apoptosis [11]. Still other payloads are also capable of killing nearby non-targeted cells through a paracrine effect, a process that is not dependent on the expression of target antigens on the cell surface. It follows that payloads are the ultimate effector components of ADCs, which mainly include: microtubule inhibitors (MMAE, MMAF, DM1, DM4), DNA damaging agents (Calicheamicin, PBD, Duocarmycin), and topoisomerase inhibitors (SN38, DXd). In addition, ADCs can further enhance tumor cell killing through mAb-partially mediated effector functions such as antibody-dependent cytotoxicity (ADCC), antibody-dependent cell phagocytosis (ADCP) and complement-dependent cytotoxicity (CDC) [12].

ADCs kill tumor cells while minimizing damage to other normal organs. This strategy not only enhances the therapeutic efficacy of the drug, but also improves the immunogenicity of the tumor [13]. One of the key mechanisms by which ADCs enhance the effectiveness of ICIs is the induction of ICD. ICD is a type of programmed cell death, which differs from conventional apoptosis in that ICD occurs with the ability to release both tumor-associated antigens (TAA) and damage-associated molecular pattern (DAMP). DAMP includes exposed cell surface calreticulin (CRT) and heat shock proteins (HSP70 and HSP90), which contribute to phagocytosis to take up dying cells, as well as extracellularly released immunostimulatory factors, such as adenosine triphosphate (ATP), high mobility group box-1 (HMGB1), and cytokines [14, 15]. Tumor cells with aberrant DAMP-release pathways (e.g., autophagy and unfolded protein response) tumor cells do not respond to stimuli that trigger immunogenic cell death. Therefore, increasing the availability of specific DAMP could convert non-immunogenic cell death into ICD [16]. These molecules are recognized, taken up and processed by dendritic cells (DCs), which subsequently present them to major histocompatibility complex (MHC II) molecules, thereby activating CD8^+^ cytotoxic T cells and CD4^+^ helper T cells [17, 18].

Previous studies have shown that microtubule destabilizing compounds (e.g., vincristine) are effective in inducing phenotypic and functional maturation of DCs, whereas microtubule stabilizers, such as paclitaxel, do not have this effect. Moreover, the antitumor effects of these drugs are dependent on an intact immune system and are significantly attenuated in immunodeficient mice. Importantly, these effects were observed even in the absence of tumor cell death. As previously mentioned, DCs activate CD8^+^ cytotoxic T cells, and the function of infiltrating CD8^+^ T cells is inhibited by the involvement of the immune checkpoint molecules PD-1 and CTLA-4, so that the use of ICIs effectively releases and maintains T cell responses against tumors [14, 19, 20]. The synergistic effect of ADCs in combination with immunotherapy for antitumor therapy has also been demonstrated in relevant preclinical trials. Among them, in the clinical trials regarding ADCs with microtubule depolymerizing agents as payloads combined with ICIs for the treatment of mice that had been inoculated with MC38 tumors, 12 mice were used in each group, and a greater number of mice in the combination group (7) achieved complete tumor rejection compared to the ADCs (1) and ICIs (3) monotherapy groups [21, 22]. This largely demonstrates that treatment with ADCs in combination with ICIs is significantly more effective than monotherapy. Similarly, topoisomerase I inhibitors such as Trastuzumab deruxtecan (T-DXd) have shown the same effect [23, 24]. Previous studies have shown that three different topoisomerase I inhibitors (cisplatin, topotecan, and irinotecan) are able to enhance the tumor killing activity of T cells, and data from relevant preclinical mouse model experiments with T-DXd may also demonstrate the superiority of combination therapy [25, 26].

There are preclinical studies suggesting that ADCs can achieve long-term anti-tumor immunity by promoting the generation of memory T cells, a phenomenon supported by some in vivo findings.D’Amico et al. used HER-2-targeted ADCs with anthracycline derivatives as potent loads to treat humanized HER-2-expressing (HER-2 +) homozygous mice. The fully cured mice were subsequently reinoculated with either the original HER-2 + cancer cells or other tumor cells of a different lineage. The results showed that mice that received reinoculation of the original HER-2 + cancer cells remained in complete remission, whereas mice inoculated with tumor cells of a different lineage rapidly developed tumors [27]. Similarly, Iwata et al. observed that a homozygous mouse model that was fully responsive to T-DXd was able to completely reject the reinoculated cancer cells. In contrast, these same tumor cells grew rapidly in control mice without any pretreatment [25]. This was also demonstrated in another preclinical study of Disitamab vedotin (RC48) in combination with an immune checkpoint inhibitor. In conclusion, there is growing evidence that ADCs may produce adaptive immunity to exert anti-tumor effects [28].

## Data from clinical studies of different ADCs in combination with ICIs

In conjunction with the strong preclinical rationale for the use of ADCs and ICIs agents in the treatment of cancer, many clinical studies have been conducted aimed at evaluating the clinical feasibility, safety, and efficacy of ADCs/ICIs combinations. Table 1 summarizes the current status of clinical trials conducted on the combination of ADCs and ICIs for the treatment of solid tumors, while Table 2 includes clinical trials for which all or part of the efficacy data have been published to date.

**Table 1:** Summary of clinical trials of ADCs in Combination with Immune Checkpoint Inhibitors

Target	ADC	Cytotoxic payload	NCT number	Phase	ICI
HER-2	Trastuzumab emtansine	DM1	NCT02924883(KATE2)	II	Atezolizumab
			NCT03032107	Ib	Pembrolizumab
			NCT04873362 (ASTEFANIA)	III	Atezolizumab
			NCT04740918 (KATE3)	III	Atezolizumab
			NCT04632992 (MyTACTIC)	II	Atezolizumab
			NCT02605915	Ib	Atezolizumab
	Trastuzumab deruxtecan	Dxd	NCT04686305 (DESTINY-Lung03)	Ib	Durvalumab
			NCT04556773 (DESTINY-Breast08)	Ib	Durvalumab
			NCT04538742 (DESTINY-Breast07)	Ib/II	Durvalumab
			NCT04379596 (DESTINY-Gastric03)	Ib/II	Durvalumab or Pembrolizumab
			NCT04042701(DS8201-A-U106)	Ib	Pembrolizumab
			NCT03742102(Begonia)	Ib/II	Durvalumab
			NCT03523572(DS8201-A-U105)	Ib	Nivolumab
			NCT03334617(HUDSON)	II	Durvalumab
			NCT04784715 (DESTINY-Breast09)	III	Pertuzumab
	Disitamab vedotin	MMAE	NCT04280341(RC48-C013)	I	Toripalimab
			NCT04264936(RC48-C014)	Ib/II	Toripalimab
			NCT05113459(RC48-C018)	II	Sintilimab
			NCT05297552(RC48-C017)	II	Toripalimab
TROP2	Sacituzumab govitecan	SN-38	NCT04863885	I/II	Ipilimumab and Nivolumab
			NCT04468061	II	Pembrolizumab
			NCT04448886(Saci-IO HR +)	II	Pembrolizumab
			NCT04434040(ASPRIA)	II	Atezolizumab
			NCT04230109(NeoSTAR)	II	Pembrolizumab
			NCT03971409(InCITe)	II	Avelumab
			NCT03547973(TROPHY U-01)	II	Pembrolizumab
			NCT03424005(Morpheus-TNBC)	Ib/II	Atezolizumab
			NCT03337698(Morpheus Lung)	Ib/II	Atezolizumab
			NCT03869190(Morpheus-UC)	Ib/II	Atezolizumab
			NCT05186974(EVOKE-02)	II	Pembrolizumab
			NCT05609968(EVOKE-03)	III	Pembrolizumab
			NCT05633667(VELOCI-TY-Lung)	II	Zimberelimab
	Datopotomab deruxtecan	Dxd	NCT04612751(TROPION-Lung04)	Ib	Durvalumab
			NCT04526691(TROPION-Lung02)	Ib	Pembrolizumab
			NCT03742102(Begonia)	Ib/II	Durvalumab
			NCT05215340(TROPION-Lung08)	III	Pembrolizumab
			NCT05687266(AVANZAR)	III	Durvalumab and Pembrolizumab
			NCT05555732(TROPION-Lung07)	III	Pembrolizumab
	SKB264	SN-38	NCT06448312	III	Pembrolizumab
			NCT06706219	III	Pembrolizumab
			NCT06711900	III	Pembrolizumab
			NCT05351788	II	KL-A167
Nectin-4	Enfortumab vedotin	MMAE	NCT04960709(VOLGA)	III	Durvalumab
			NCT04700124(KEYNOTE-B15)	III	Pembrolizumab
			NCT04223856(EV-302)	III	Pembrolizumab
			NCT03924895(KEYNOTE-905/EV-303)	III	Pembrolizumab
			NCT03606174	II	Pembrolizumab and Nivolumab
			NCT03288545(EV-103)	I/II	Pembrolizumab
			NCT03869190(Morpheus-UC)	Ib/II	Atezolizumab
			NCT05239624(EV-ECLIPSE)	II	Pembrolizumab
			NCT04225117(EV-202)	II	Pembrolizumab
FLOR1 (FRα)	Mirvetuximab soravtansine	DM4	NCT03835819	II	Pembrolizumab
			NCT02606305	Ib/II	Pembrolizumab
TF	Tisotumab vedotin	MMAE	NCT03786081	Ib/II	Pembrolizumab
			NCT03485209(innovaTV 207)	II	Pembrolizumab
B7-H3	Vobramitamab duocarmazine(MGC018)		NCT03729596	I/II	Retifanlimab
	Enoblituzumab		NCT02475213	I	Pembrolizumab
LIV-1	Ladiratuzumab vedotin	MMAE	NCT03310957(SGNLVA-002)	Ib/II	Pembrolizumab
			NCT03424005 (Morpheus-TNBC)	Ib/II	Atezolizumab
			NCT02099058	I/Ib	Nivolumab
CEACAM5	Tusamitamab ravtansine	DM4	NCT04524689 (CARMEN-LC05)	II	Pembrolizumab
			NCT04394624 (CARMEN-LC04)	II	Pembrolizumab
Claudin18.2	LaNova Medicines-302	MMAF	NCT05188664	I/II	Toripalimab
			NCT05934331	II	Toripalimab
			NCT05994001	Ib/II	Cardonilizumab

Target	ICI type	Condition	Setting	Primary endpoint	Status
HER-2	PD-L1 inhibitor	HER2-positive Breast Cancer	Locally Advanced or Metastatic	PFS,AEs	Completed
	PD-1 inhibitor	HER2-positive Breast Cancer	Metastatic	AEs	Completed
	PD-L1 inhibitor	HER2-positive Breast Cancer	High Risk of Recurrence Following Preoperative Therapy	IDFS	Active, not recruiting
	PD-L1 inhibitor	HER2-positive and PD-L1-positive Breast Cancer	Locally Advanced or Metastatic	PFS,OS	Terminated
	PD-L1 inhibitor	HER2-positive Solid Tumors	Advanced Unresectable or Metastatic	ORR	Completed
	PD-L1 inhibitor	HER2-positive Breast Cancer	Locally Advanced or Metastatic	DLT,AEs	Completed
	PD-L1 inhibitor	HER2-oe Non-Small Cell Lung Cancer	Locally Advanced or Metastatic	AEs,SAEs	Recruiting
	PD-L1 inhibitor	HER2-low Breast Cancer	Metastatic	AEs,SAEs	Active, not recruiting
	PD-L1 inhibitor	HER2-positive Breast Cancer	Advanced or Metastatic	AEs,SAEs	Active, not recruiting
	PD-L1 inhibitor or PD-1 inhibitor	HER2-positive esophageal/gastric/Gastric and Esophagogastric Junction Adenocarcinoma	Advanced or Metastatic	AEs,SAEs,DLT,ORR	Recruiting
	PD-1 inhibitor	HER2-expressing Breast Cancer and HER2-expressing or HER2-mutant Non-Small Cell Lung Cancer	Advanced or Metastatic	DLT,ORR	Active, not recruiting
	PD-L1 inhibitor	Triple-Negative Breast Cancer	Advanced Unresectable or Metastatic	AEs,ORR	Active, not recruiting
	PD-1 inhibitor	HER2-expressing Breast Cancer and Urothelial Cancer	Advanced or Metastatic	ORR	Completed
	PD-L1 inhibitor	Non-Small Cell Lung Cancer	Metastatic	ORR	Active, not recruiting
		HER2-positive Breast Cancer	Metastatic	PFS	Active, not recruiting
	PD-1 inhibitor	HER2-positive Solid tumors	Advanced or Metastatic	DLT,AEs	Recruiting
	PD-1 inhibitor	Urothelial Cancer	Advanced or Metastatic	AEs	Unknown status
		HER2-positive Gastric and Esophagogastric Junction Adenocarcinoma	Locally Advanced	PCR	Unknown status
	PD-1 inhibitor	Muscle Invasive Bladder Cancer		PCR	Recruiting
TROP2	CTLA-4 inhibitor and PD-1 inhibitor	Urothelial Cancer	Metastatic	MTD,ORR	Active, not recruiting
	PD-1 inhibitor	Triple-Negative Breast Cancer	Metastatic	PFS	Recruiting
	PD-1 inhibitor	HR + /HER2- Breast Cancer	Invasive or Metastatic	PFS	Active, not recruiting
	PD-L1 inhibitor	Triple-Negative Breast Cancer		Rate of undetectable circulating tumor cfDNA- 6 Cycles	Active, not recruiting
	PD-1 inhibitor	Triple-Negative Breast Cancer	Localized	PCR	Recruiting
	PD-L1 inhibitor	Triple-Negative Breast Cancer	Stage IV or Unresectable, Recurrent	BORR	Recruiting
	PD-1 inhibitor	Urothelial Cancer	Unresectable Locally Advanced/Metastatic	ORR,PFS,TEAEs	Recruiting
	PD-L1 inhibitor	Breast Cancer	Metastatic	ORR	Recruiting
	PD-L1 inhibitor	Non-Small Cell Lung Cancer	Metastatic	ORR	Active, not recruiting
	PD-L1 inhibitor	Urothelial Cancer and Bladder Cancer	Advanced or Metastatic	ORR,PCR	Active, not recruiting
	PD-1 inhibitor	Non-Small Cell Lung Cancer	Advanced or Metastatic	ORR,DLT	Active, not recruiting
	PD-1 inhibitor	Non-Small Cell Lung Cancer	Metastatic	PFS,OS	Recruiting
	PD-1 inhibitor	Non-Small Cell Lung Cancer	Resectable,Advanced or Metastatic	ORR	Recruiting
	PD-L1 inhibitor	Non-Small Cell Lung Cancer	Advanced or Metastatic	DLT,TEAEs	Recruiting
	PD-1 inhibitor	Non-Small Cell Lung Cancer	Advanced or Metastatic	DLT,TEAEs	Active, not recruiting
	PD-L1 inhibitor	Triple-Negative Breast Cancer	Advanced or Metastatic	AEs,ORR	Active, not recruiting
	PD-1 inhibitor	Non-Small Cell Lung Cancer	Metastatic	PFS,OS	Recruiting
	PD-L1 inhibitor and PD-1 inhibitor	Non-Small Cell Lung Cancer	Advanced or Metastatic	PFS,OS	Active, not recruiting
	PD-1 inhibitor	PD-L1 TPS < 50% Non-Small Cell Lung Cancer	Advanced or Metastatic	PFS,OS	Recruiting
	PD-1 inhibitor	PD-L1 Positive Non-Small Cell Lung Cancer	Advanced or Metastatic	PFS	Recruiting
	PD-1 inhibitor	Non-Small Cell Lung Cancer	Unresectable stage III	18 m EFS rate	Not yet recruiting
	PD-1 inhibitor	Non-Squamous Non-Small Cell Lung Cancer	Advanced or Metastatic	PFS	Recruiting
	CD47 inhibitor	Non-Small Cell Lung Cancer	Advanced or Metastatic	AEs,ORR	Recruiting
Nectin-4	PD-L1 inhibitor	Muscle Invasive Bladder Cancer		AEs	Active, not recruiting
	PD-1 inhibitor	Muscle Invasive Bladder Cancer		EFS	Active, not recruiting
	PD-1 inhibitor	Urothelial Cancer	Advanced or Metastatic	PFS,OS	Active, not recruiting
	PD-1 inhibitor	Muscle Invasive Bladder Cancer		EFS between groups C and B	Active, not recruiting
	PD-1 inhibitor	Urothelial Cancer	Advanced or Metastatic	ORR	Terminated
	PD-1 inhibitor	Urothelial Cancer	Advanced or Metastatic	AEs,ORR,PCR	Active, not recruiting
	PD-L1 inhibitor	Urothelial Cancer	Advanced or Metastatic	ORR,PCR	Active, not recruiting
	PD-1 inhibitor	Urothelial Cancer	Neoadjuvant	PCR	Recruiting
	PD-1 inhibitor	Malignant Solid tumors	Advanced or Metastatic	ORR	Active, not recruiting
FLOR1 (FRα)	PD-1 inhibitor	FRα positive,MSS Endometrial cancer	Recurrent or Persistent	ORR,PFS	Active, not recruiting
	PD-1 inhibitor	FRα positive Epithelial ovarian cancer, primary peritoneal cancer, or fallopian tube cancer	Advanced	TEAEs,ORR	Completed
TF	PD-1 inhibitor	Cervical Cancer	Recurrent or Stage IVB	DLT,ORR	Active, not recruiting
	PD-1 inhibitor	Solid tumors	Advanced or Metastatic	ORR	Active, not recruiting
B7-H3	PD-1 inhibitor	Solid tumors	Advanced or Metastatic	DLT,AEs,SAEs	Terminated
	PD-1 inhibitor	Solid tumors	Advanced	AEs,SAEs	Completed
LIV-1	PD-1 inhibitor	Triple-Negative Breast Cancer	Locally Advanced or Metastatic	ORR,DLT,AEs	Completed
	PD-L1 inhibitor	Breast Cancer	Metastatic	ORR	Recruiting
	PD-1 inhibitor	Solid tumors	Advanced	AEs,RPTD,AUC,Cmax,Tmax	Active, not recruiting
CEACAM5	PD-1 inhibitor	CEACAM5-positive Non-Small Cell Lung Cancer	Advanced or Metastatic	DLT,ORR	Terminated
	PD-1 inhibitor	CEACAM5-positive Non-Small Cell Lung Cancer	Metastatic	DLT,ORR	Terminated
Claudin18.2	PD-1 inhibitor	Solid tumors	Advanced	DLT,RP2D,OBD,MTD	Completed
	PD-1 inhibitor	Gastro-Intestinal Cancer	Advanced	PFS	Recruiting
	TIGIT inhibitor	Claudin 18.2 Positive Bile Duct Cancer	Advanced	ORR,AEs	Recruiting

**Table 2:** Clinical efficacy of ADCs in Combination with Immune Checkpoint Inhibitors

Target	ADC	NCT number	Phase	ICIs	whether arrived primary endpoints
HER-2	Trastuzumab emtansine	NCT02924883(KATE2)	II	Atezolizumab	Yes
		NCT03032107	Ib	Pembrolizumab	Yes
		NCT02605915	Ib	Atezolizumab	Yes
	Trastuzumab deruxtecan	NCT04379596 (DESTINY-Gastric03)	Ib/II	Durvalumab or Pembrolizumab	Yes
		NCT04042701 (DS8201-A-U106)	Ib	Pembrolizumab	Yes
		NCT03523572 (DS8201-A-U105)	Ib	Nivolumab	Yes
		NCT03334617 (HUDSON)	II	Durvalumab	Yes
	Disitamab vedotin	NCT04280341 (RC48-C013)	I	Toripalimab	Yes
		NCT04264936(RC48-C014)	Ib/II	Toripalimab	Yes
Trop2	Sacituzumab govitecan	NCT04448886 (Saci-IO HR +)	II	Pembrolizumab	Yes
		NCT03424005 (Morpheus-TNBC)	Ib/II	Atezolizumab	Yes
		NCT05186974 (EVOKE-02)	II	Pembrolizumab	Yes
	Datopotomab deruxtecan	NCT04526691(TROPION-Lung02)	Ib	Pembrolizumab	Yes
		NCT03742102(Begonia)	Ib/II	Durvalumab	Yes
	SKB264	NCT05351788	II	KL-A167	Yes
Nectin-4	Enfortumab vedotin	NCT04223856(EV-302)	III	Pembrolizumab	Yes
		NCT03288545(EV-103)	I/II	Pembrolizumab	Yes
		NCT04225117(EV-202)	II	Pembrolizumab	Yes
FLOR1 (FRα)	Mirvetuximab soravtansine	NCT03835819	II	Pembrolizumab	No
TF	Tisotumab vedotin	NCT03786081	Ib/II	Pembrolizumab	Yes
B7-H3	Enoblituzumab	NCT02475213	I	Pembrolizumab	Yes
LIV-1	Ladiratuzumab vedotin	NCT02099058	I/Ib	Nivolumab	Yes
CEACAM5	Tusamitamab ravtansine	NCT04524689 (CARMEN-LC05)	II	Pembrolizumab	Yes
Claudin18.2	LaNova Medicines-302	NCT05994001	Ib/II	Cardonilizumab	No

Target	Efficacy data				
	ORR, %	mOS, months	mPFS, months	mDOR, months	DCR(%)
HER-2	Atezolizumab:45 Placebo:43	NA	Atezolizumab:8.2 Placebo:6.8	NA	NA
	20	NA	9.6	10.1	60
	Triple:14 Duplex:35 Quadruple:100	NA	NA	NA	NA
	GroupD:58 GroupE:63 GroupF:13	NA	NA	NA	NA
	HER2 e:54.5 HER2 m:66.7	NA	HER2 e:15.1 HER2 m:11.3	HER2 e:20.2 HER2 m:15.1	NA
	mBC:HER2 + :65.6; HER2 low:50.0 mUC:HER2 high:36.7; HER2 low:NA	mBC:HER2 + :NA; HER2 low:19.5 mUC:HER2 high:11.0; HER2 low:NA	mBC:HER2 + :11.6; HER2 low:7.0 mUC:HER2 high:6.9; HER2 low:NA	mBC:HER2 + :N; HER2 low:5.5 mUC:HER2 high:13.1; HER2 low:NA	NA
	HER2 e:26.1 HER2 m:35	HER2 e:9.5 HER2 m:10.6	HER2 e:2.8 HER2 m:5.7	NA	NA
	G/GEJ:43 Other solid tumor:25	G/GEJ:16.8 Other solid tumor:10.5	G/GEJ:6.2 Other solid tumor:4.9	G/GEJ:5.1 Other solid tumor:8.2	G/GEJ:75 Other solid tumor:75
	73.2	33.1	9.3	8.6	90.2
Trop2	GroupA(combination):21.2 GroupB(SG):17.3	GroupA(combination):16.9 GroupB(SG):17.1	GroupA(combination):8.4 GroupB(SG):6.2	NA	NA
	Sacituzumab govitecan + Atezolizumab:76.7 control:66.7	NA	Sacituzumab govitecan + Atezolizumab:12.2 control:6.9	Sacituzumab govitecan + Atezolizumab:14.0 control:7.1	Sacituzumab govitecan + Atezolizumab:93.3 control:100
	squamous: CohortA:73 CorhortB:54 non-squamous: CohortA:67 CorhortB:37	NA	NA	NA	squamous: CohortA:82 CorhortB:85 non-squamous: CohortA:89 CorhortB:74
	Duplex:52 Triple:56	NA	Duplex:11.1 Triple:6.8	Duplex:NA Triple:12.9	Duplex:88 Triple:89
	79	NA	13.8	15.5	NA
	1A:48.6 1B:77.6	NA	1A:15.4 1B:NA	NA	1A:94.6 1B:100
Nectin-4	29.1	31.5	12.5	NA	NA
	73.3	26.1	12.3	25.6	93.3
	23.9	6	3.9	9.4	56.5
FLOR1 (FRα)	37.5	NA	NA	NA	NA
TF	GroupE:40.6 GroupF:35.3	GroupE:NA GroupF:15.3	GroupE:5.3 GroupF:5.6	GroupE:NA GroupF:14.1	GroupE:81.3 GroupF:73.5
B7-H3	HNSCC:33.3 NSCLC:35.7 UC:5.9 Melanoma:7.7	HNSCC:17.38 NSCLC:12.32 UC:5.72 Melanoma:14.19	HNSCC:3.48 NSCLC:4.83 UC:2.18 Melanoma:2.07	NSCLC:8.3	NA
LIV-1	30.6	NA	5.9	NA	86.1
CEACAM5	52.6	NA	11.6	12.4	NA
Claudin18.2	NA	NA	NA	NA	NA

### HER-2

HER-2 is a key member overexpressed in a variety of cancers, including breast cancer, urothelial carcinoma [29], colorectal cancer [30], and non-small cell lung cancer [31], etc. Activated HER-2 promotes tumor growth and spread [32]. Therefore, HER-2 is a promising therapeutic target for the treatment of many HER-2-positive tumors [33, 34].

Trastuzumab emtansine (T-DM1) was the first ADC approved for solid tumors. Results from a number of previous combination trials have shown higher objective remission rates (ORR) in PD-L1-positive patients, as well as a more significant efficacy advantage [18, 35, 36]. However, it is worth noting that T-DM1 combination immunotherapy did not show significant clinical significance in a trial in which all patients were PD-L1 negative [37].

T-DXd is currently a hot research topic for the treatment of HER-2-positive solid tumors. Several trials are exploring the feasibility and potential efficacy of combining this ADC with immunotherapy. In the breast cancer (BC) portion of the phase Ib DS8201-A-U105 trial, 45 HER-2-positive BC patients were treated with T-DXd in combination with Nivolumab. Interim analysis showed that HER-2-positive patients had an ORR of 65.6% and an mPFS of 11.6 m, while patients with low HER-2 expression had an ORR of 50.0% and an mPFS of 7.0 m. Patients with HER-2 high-expression (IHC 3 + /2 +) mUC who received the combination treatment in the uroepithelial carcinoma (UC) portion of the same trial achieved ORR of 36.7%, mPFS of 6.9 m and mOS of 11.0 m. The results demonstrated that the recommended extended dose (RDE) was well tolerated and showed significant antitumor activity in patients with HER-2-positive mBC and HER-2-overexpressing mUC who had received extensive pretreatment. This antitumor activity is consistent with previous studies of T-DXd monotherapy [38–42]. The ongoing HUDSON trial evaluated the antitumor efficacy of Durvalumab in combination with T-DXd for the treatment of patients with HER-2 overexpressing (HER-2e) or HER-2-mutant (HER-2m) NSCLC. Patients with HER-2 m demonstrated significant antitumor activity in terms of ORR (35.0% vs. 26.1%), PFS (5.7 vs 2.8 m), and OS (10.6 vs 9.5 m) were superior to HER-2e patients, suggesting that combination therapy demonstrated a trend toward more significant efficacy in HER-2m patients [43]. In addition, the DS8201-A-U106 study evaluated the anti-tumor effects of Pembrolizumab in combination with T-DXd in HER-2e and HER-2m NSCLC patients. The latest interim analysis showed that patients in both cohorts demonstrated significant antitumor activity (ORR: 54.5% vs 66.7%; mPFS: 15.1 vs 11.3 m) [44]. A comparison of the results of the HUDSON trial and the DS8201-A-U106 study reveals that the efficacy of T-DXd in combination with a PD-1 inhibitor is significantly more prominent in the same treatment population. Trials are also evaluating the efficacy of T-DXd in combination with immunotherapy (Module 1 of DESTINY-Breast07) as well as triple therapy with T-DXd, immunotherapy and chemotherapy (Module 4 of DESTINY-Breast07) in patients with HER-2-positive mBC [45]. The DESTINY-Gastric03 trial on the treatment of patients with advanced/metastatic HER-2 + esophageal, gastric or gastroesophageal junction adenocarcinoma (GEJA) is also ongoing [46].

RC48-C013, a evaluating RC48 in combination with Phase I trial for the treatment of HER-2-positive advanced gastric cancer or gastroesophageal junction (G/GEJ) and other solid tumors, achieved an ORR of 50.0% in patients receiving the recommended Phase II dose (RP2D), which was significantly higher than that of the 24.8% in patients treated with RC48 alone. Even in G/GEJ patients with low HER-2 expression, the ORR reached 46.0%. Further analysis showed that the efficacy of primary patients was superior to that of patients who had received prior anti-HER-2 therapy, with significant improvements in ORR (53% vs 43%), mPFS (6.1 vs 4.2 m) and mOS (24.7 vs 11.9 m). In addition, patients who received RP2D with PD-L1 CPS ≥ 1 demonstrated higher ORR and longer PFS and OS. This suggests that RC48 combination immunotherapy could improve the efficacy of some G/GEJ patients, which is clinically important ICIs [47, 48]. RC48-C014 is another phase Ib/II trial for la/mUC, and the most recent analysis of the data showed that, using the same treatment combination The mOS of patients was 33.1 m, mPFS was 9.3 m and ORR was as high as 73.0%, demonstrating significant efficacy and OS advantage in long-term follow-up [49].

### Trop-2

In recent years, it has been found that the expression level of (Trop-2) is significantly increased in a variety of solid tumors. This upregulation not only promotes the proliferation, growth and spread of tumor cells, but is also closely associated with poorer prognosis and higher risk of metastasis human trophoblast surface antigen [50, 51].

Sacituzumab govitecan (SG) is the first Trop 2-targeted ADC. Previously treated triple-negative and hormone receptor-positive/HER-2-negative (HR + /HER-2-) mBC patients were evaluated in the Saci-IO HR + trial on SG. In PD-L1-positive (CPS ≥ 1) HR + /HER-2- patients, SG in combination with Pembrolizumab (Group A) showed superiority over single agent (Group B): median PFS was prolonged by 4.4 m (11.1 vs 6.7 m) and median OS was prolonged by 6 m (18.5 vs 12.5 m), and the experimental results were more supportive of the combination, however, the results of the trial were more favorable to the combination, however, the different PD -L1 expression tiers (CPS ≥ 1 vs CPS < 1) showed no significant difference in PFS and OS [52]. In addition, in the Morpheus-TNBC trial, SG in combination with Atezolizumab showed a trend toward benefit (12.2 vs 5.9 m) despite immature PFS data [53]. Comparison of the efficacy data in the combination therapy portion of the above two trials showed that the antitumor activity of SG combined with Atezolizumab was significantly better than that of SG combined with Pembrolizumab, with ORR of 76.7 and 21.2%, respectively. In addition, SG in combination with Atezolizumab also demonstrated superior efficacy in terms of median PFS (12.2 vs 8.4 m). The most recent data from the Phase II EVOKE-02 study indicate that, regardless of histologic type (squamous or non-squamous), SG in combination with Pembrolizumab demonstrated antitumor activity in untreated mNSCLC demonstrated anti-tumor activity in [54].

Datopotomab deruxtecan (Dato) is a Trop-2-targeted ADC with a topoisomerase I inhibitor as its payload. In the BEGONIA trial, patients with metastatic triple-negative breast cancer (mTNBC) who were treated with Dato in combination with durvalumab achieved an ORR of 79.0% (6 out of 62 patients) complete remission (CR), 43 partial remission (PR)) [55]. Data from another phase Ib trial of Dato in combination with Pembrolizumab (± platinum analog) for NSCLC showed durable antitumor activity in patients in any PD-L1 expression state with both double and triple therapy [56]. Another Trop-2-targeted ADC drug, SKB264, also showed promising antitumor efficacy results (ORR of 48.6%) in patients with primary advanced NSCLC [57]. Several other Phase III clinical trials of SKB264 are ongoing and data are not yet available.

### Nectin-4

Enfortumab vedotin (EV), an ADC targeting Nectin-4, has been approved as a single agent for the treatment of platinum and ICIs-refractory advanced uroepithelial cancer (aUC). In the Phase III EV-302 trial, EV in combination with Pembrolizumab demonstrated significant efficacy in patients with previously untreated a/mUC, with an mPFS of 12.5 m, which was significantly better than that of the chemotherapy group at 6.3 m, and an mOS of 31.5 m, which was almost twice that of the chemotherapy group. In addition, the ORR of the combination therapy was also significantly higher than that of the chemotherapy group (67.7% vs. 44.4%), and reduced the risk of disease progression by 55% and the risk of death by 53%. These results suggest that combination therapy with EV offers significant and clinically meaningful advantages over conventional chemotherapy in the first-line treatment of a/mUC [58]. Moreover, in the phase II EV-202 study, the confirmed ORR of EV monotherapy in patients with head and neck cancer (HNC) was 23.9%, which exceeded the prespecified threshold (17.5%) and showed good anti-tumor activity. This provides a basis for evaluating EV in combination with Pembrolizumab in patients with HNC. Another cohort of EV-202 is currently investigating EV in combination with Pembrolizumab as a first-line treatment for patients with recurrent/metastatic head and neck squamous carcinoma (R/M HNSCC) with PD-L1 CPS ≥ 1 [59].

### Other new targets

Previous studies demonstrated that Mirvetuximab soravtansine (MIRV) showed well tolerability and single-agent activity in a Phase I dose extension study in patients with FRα-positive advanced or recurrent endometrial cancer (EC). A subsequent Phase II study evaluated its efficacy in combination with Pembrolizumab. Of the 16 patients treated, the ORR was 37.5%, including 1 patient got CR and 5 patients got PR. Recent findings suggest that MIRV in combination with Pembrolizumab meets the primary endpoint in the treatment of FRα-positive recurrent microsatellite-stabilized (MSS/pMMR) plasmacytoid EC, with value for further study, and studies targeting subgroups of potential benefit are also ongoing [60]. In addition, Tisotumab vedotin (TV) is a kind of ADCs that targets tissue factor (TF). In a Phase Ib/II study in recurrent/metastatic cervical cancer (r/mCC), TV demonstrated promising anti-tumor activity when used in combination with bevacizumab, Pembrolizumab, or carboplatin for first- or second- or third-line treatment. This provides support for further exploration of TV in combination with single agents. Expansion studies of TV in combination with carboplatin, Pembrolizumab, and optionally in combination with bevacizumab for first-line treatment are currently underway [61]. Finally, the regimen of Tusamitamab ravtansine (ADC targeting CEACAM5) in combination with Pembrolizumab showed positive preliminary results in a phase I b/II trial in advanced non-squamous NSCLC, with an ORR of 47.8%, an mPFS of 11.6 m, and an mDOR of 12.4 m [62]. In addition to this, there are several drugs targeting Claudin 18.2 (mainly in GI tumors) currently in clinical trials. One of these Phase Ib/II clinical trials was announced at ASCO: out of 6 patients whose efficacy could be evaluated, 3 achieved PR, so its BOR in Phase Ib was 50% [63].

## Safety and re-challenge of ADCs in combination with ICIs

### Drug safety and adverse reactions

Table 3 summarizes the safety data from the clinical trials in Table 2.

**Table 3:** Safety of Trials of ADCs in Combination with Immune Checkpoint Inhibitors

Target	ADC	NCT number	Phase	ICI	Safety
HER-2	Trastuzumab emtansine	NCT02924883(KATE2)	II	Atezolizumab	Grade ≥ 3 AEs:thrombocytopenia, increased aspartate aminotransferase, anemia, neutropenia and increased alanine aminotransferase
		NCT03032107	Ib	Pembrolizumab	All-Grade AEs:fatigue, anemia, elevated aspartate aminotransferase, constipation, nausea, and pneumonitis Any Grade 3:fatigue, elevated aspartate aminotransferase, elevated alanine aminotransferase, pneumonitis, lung infection, oral mucositis, and vomiting
		NCT02605915	Ib	Atezolizumab	The most common AEs: fatigue, diarrhea, and nausea
	Trastuzumab deruxtecan	NCT04379596 (DESTINY-Gastric03)	Ib/II	Durvalumab or Pembrolizumab	ILD/pneumonitis: GroupD:8 patients; GroupE:5 patients; Grade ≥ 3 AEs: GroupD:91%; GroupE: 81%; GroupF: 28%;
		NCT04042701(DS8201-A-U106)	Ib	Pembrolizumab	ILD/pneumonitis:11 patients Grade ≥ 3 TEAEs:38.2% Any TEAEs:94.5%
		NCT03523572(DS8201-A-U105)	Ib	Nivolumab	mBC:HER2 + :Grade ≥ 3 TEAEs: nausea,fatigue and constipation HER2-low:Grade ≥ 3 TEAEs:nausea,vomiting and anemia mUC:HER2-high:nausea, fatigue and vomiting HER2-low:nausea and fatigue TEAEs leading to discontinuation:ILD/pneumonitis
		NCT03334617(HUDSON)	II	Durvalumab	Grade ≥ 3 TEAEs:pneumonitis,pulmonary embolism,anemia
	Disitamab vedotin	NCT04280341 (RC48-C013)	I	Toripalimab	Any TRAEs:a decreased white blood cell count,a decreased neutrophil count,asthenia,an increased AST level,and an increased ALT level Grade ≥ 3 TRAEs:a decreased neutrophil count and a decreased white blood cell count
		NCT04264936(RC48-C014)	Ib/II	Toripalimab	Grade ≥ 3 TRAEs:gamma-glutamyltransferase increased,asthenia,hypertriglyceridaemia,and alanine aminotransferase increased,blood glucose increased,pneumonitis,aspartate aminotransferase increased,blood creatine phosphokinase increased,and neutropenia
Trop2	Sacituzumab govitecan	NCT04448886(Saci-IO HR +)	II	Pembrolizumab	Grade ≥ 2 AEs: GroupA:neutropenia,fatigue,alopecia,anemia,leukopenia,,diarrhea and nausea GroupB:neutropenia,alopecia,diarrhea,nausea,fatigue and anemia
		NCT03424005 (Morpheus-TNBC)	Ib/II	Atezolizumab	Grade 3–4 AEs: Sacituzumab govitecan + Atezolizumab:70% control:44.4% SAEs: Sacituzumab govitecan + Atezolizumab:23.3% control:44.4%
		NCT05186974(EVOKE-02)	II	Pembrolizumab	Grade ≥ 3 TEAEs:70%
	Datopotomab deruxtecan	NCT04526691 (TROPION-Lung02)	Ib	Pembrolizumab	All grade TEAE:stomatitis and nausea Grade ≥ 3 TEAEs:Duplex:57%,Triple:76%
		NCT03742102 (Begonia)	Ib/II	Durvalumab	The most common AEs:Nausea and stomatitis Grade 3–4 AEs:57% SAEs:23%
	SKB264	NCT05351788	II	KL-A167	Grade ≥ 3 TRAEs:neutrophil count decreased, white blood cell count decreased, anemia, rash and drug eruption
Nectin-4	Enfortumab vedotin	NCT04223856 (EV-302)	III	Pembrolizumab	Any Grade AEs:peripheral sensory neuropathy, pruritus,and alopecia; Grade ≥ 3 TRAEs:maculopapular rash, hyperglycemia,and neutropenia
		NCT03288545 (EV-103)	I/II	Pembrolizumab	The most common TRAEs:peripheral sensory neuropathy, fatigue, and alopecia Grade ≥ 3 TRAEs:asymptomatic lipase elevation, fatigue, and maculopapular rash
		NCT04225117(EV-202)	II	Pembrolizumab	The most common TRAEs:alopecia, fatigue, and peripheral sensory neuropathy Grade ≥ 3 TRAEs:anemia and decreased neutrophil count
FLOR1 (FRα)	Mirvetuximab soravtansine	NCT03835819	II	Pembrolizumab	The most common TRAEs:AST elevation, blurred vision, fatigue and diarrhea
TF	Tisotumab vedotin	NCT03786081	Ib/II	Pembrolizumab	Grade ≥ 3 TRAEs: GroupE:anemia,asthenia,hypokalemia,acute kidney injury GroupF:anemia,intetinal obstruction,weight decreased,acute kidney injury
B7-H3	Enoblituzumab	NCT02475213	I	Pembrolizumab	The most common TRAEs:infusion-related reactions (IRRs),fatigue Grade ≥ 3 TRAEs:IRRs,increased lipase
LIV-1	Ladiratuzumab vedotin	NCT02099058	I/Ib	Nivolumab	Any Grade AEs: peripheral sensory neuropathy, dermatitis acneiform, diarrhea,and hypoalbuminemia Grade ≥ 3 AEs:were pulmonary embolism (PE), hypokalemia,and diarrhea, malignant neoplasm progression, peripheral sensory neuropathy, and hypophosphatemia Grade ≥ 3 TRAEs:hypophosphatemia and peripheral sensory neuropathy
CEACAM5	Tusamitamab ravtansine	NCT04524689(CARMEN-LC05)	II	Pembrolizumab	SAE:36.8% Grade ≥ 3 TEAEs:64.9%
Claudin18.2	LaNova Medicines-302	NCT05994001	Ib/II	Cardonilizumab	The most common TRAEs:bilirubin increase, thrombocytopenia, neutropenia, infusion reactions (rash), leukopenia (42.9%), anemia

In non-randomized early clinical trials of T-DM1 combination immunotherapy, the most common adverse events (AEs) included fatigue, anemia, nausea, and pneumonia. In several trials, some patients experienced grade 3 or more serious adverse events such as thrombocytopenia, elevated aspartate aminotransferase (AST), elevated alanine aminotransferase (ALT), and pneumonia. Discontinuation of treatment due to adverse events was more frequent in patients receiving T-DM1 in combination with Atezolizumab than T-DM1 alone, and one death due to phagocytosis syndrome occurred in the experimental group [35]. When T-DM1 was combined with Pembrolizumab, four patients developed after cycles 2, 3, 4, and 12 of treatment with pneumonia, all of which were suspected to be ICIs-associated pneumonia and improved after receiving steroid therapy [18]. In contrast, in the randomized KATE 2 trial, despite more discontinuations due to pneumonia in the Atezolizumab group, the incidence of all grades and grade 3 or worse pneumonia was similar in both groups, suggesting that combining Atezolizumab does not significantly increase the risk of pneumonia [35].

Several trials have shown that patients treated with T-DXd combination immunotherapy are more likely to experience serious adverse events (SAEs) compared to single-agent T-DXd. Preliminary results from the DESTINY-Gastric03 trial showed a higher incidence of Grade ≥ 3 AEs with triple therapy with T-DXd (91%) than with two-agent therapy (81%) and single-agent therapy (64%) [46]. In the mBC and mUC modules of the DS8201-A-U105 trial, the most common treatment-related adverse events (TEAEs) were nausea, fatigue, and vomiting. Compared to patients with low HER-2 expression, HER-2-positive patients had a higher incidence and severity of AEs, particularly interstitial lung disease (ILD)/pneumonia, with some patients experiencing grade 5 ILD/pneumonia, leading to discontinuation or death [38–42]. A similar situation was observed in the phase Ib trial in combination with Pembrolizumab and in the HUDSON trial. In that trial, pneumonia was the most common grade ≥ 3 AE. Management of ILD/pneumonia consisted mainly of drug discontinuation and steroid therapy, with some patients improving with treatment [43].

In the two trials testing Dato, stomatitis and nausea were the most common TEAEs, with higher rates of nausea especially in two- or three-drug combinations. The incidence of stomatitis was 57.0% in the TROPION-Lung02 trial compared with 65.0% in the Begonia trial, both of which were predominantly grade 1–2 adverse events [55, 56].

Finally, in the Enfortumab vedotin in combination with Pembrolizumab trial, peripheral sensory neuropathy (PN) was the most common TEAE, mostly mild to moderate. In contrast, in the EV-103 trial, peripheral neuropathy and skin reactions were grade ≥ 3 AEs requiring special attention [58, 64].

### Re-challenge

Serious drug-related adverse reactions are a common reason for discontinuation during oncology treatment. However, most adverse reactions are usually mild and reversible, and can be alleviated by symptomatic treatment and return to normal function after discontinuation. When adverse reactions are effectively controlled, patients can continue their original treatment regimen or make dose adjustments as clinically indicated. This restarting of the original regimen after discontinuation is known as “rechallenge.”

ILD/pneumonia is a common discontinuation-related adverse event in many cancer therapies, and ADCs are no exception. In the DESTINY clinical program, T-DXd demonstrated an overall manageable safety profile, with hematologic and gastrointestinal AEs being the most common, but ILD/pneumonia being the AE of particular concern [65–69]. Grade 2 or higher ILD/pneumonia requiring permanent discontinuation of the drug and treatment with high-dose steroids. The 2024 ESMO Congress reported a retrospective analysis of T-DXd retreatment, with data from nine clinical trials covering patients with HER-2-altered breast, gastric, colorectal, and non-small-cell lung cancers who had received at least one dose of T-DXd. Of the 2,145 patients 193 developed grade 1 ILD, of which 97 were relieved by hormone therapy and 45 patients were re-treated with T-DXd after recovery. And of these 45 patients, 15 patients had recurrent ILD due to the rechallenge dose being consistent with the original dose (both grade 1 and 2). Of all the patients with recurrent ILD, only eight recovered completely. In addition, two patients were retreated before they had fully recovered from grade 1 ILD, resulting in progression of ILD to grades 2 and 3, respectively [70].

In addition, there is a case report describing a patient who underwent T-DXd rechallenge after failure of multiple lines of therapy. The patient was diagnosed with grade 3 ILD after two cycles of therapy and was treated with steroids and antibiotics. After symptoms resolved and returned to grade 1 ILD, the patient underwent T-Dxd rechallenge, but at a reduced dose, while maintaining low-dose steroid therapy. During the rechallenge period, the patient efficacy reached PR and no exacerbation or recurrence of ILD was observed [71]. In addition, in the phase 1b trial of T-DM1 in combination with Pembrolizumab, four patients with suspected ICIs-associated pneumonia improved after steroid treatment. Three of these patients permanently discontinued Pembrolizumab but continued to use T-DM1 and no significant increase in pulmonary abnormalities was observed [18].

## ADCs development and future prospects

In recent years, the rapid development of ADCs has opened up new avenues for tumor therapy. Classical ADCs targets (e.g., HER-2, CD30, and CD79b) have achieved remarkable results in the treatment of a variety of tumors. However, the problems of drug resistance and heterogeneity of these traditional targets have gradually emerged [72, 73]. And tumor resistance is usually associated with downregulation of the expression of therapeutic targets or off-target effects. For this reason, researchers have used combination target strategies or target epitope optimization to enhance tumor efficacy and delay the onset of drug resistance.

### Antibody and target selection

The ideal backbone for constructing ADCs is the ability to recognize antigens that are specifically expressed only on the surface of cancer cells and are completely absent from noncancerous tissues, thus enabling tumor-specific payload delivery. However, most ADCs targets (e.g., HER-2 and TROP-2) are also expressed to some extent in noncancerous tissues, which may lead to the development of target-dependent and non-dependent toxicity. To enhance tumor specificity, antibodies that recognize tumor-specific antigens with structural variants such as truncation, cleavage or post-translational modifications are being explored [73–75]. Among them, bispecific ADCs can significantly improve payload delivery and cytotoxicity by combining antibodies to both targets and conventional payloads. In addition, nano-antibody–drug couplings (NDCs) developed to address the permeability problem caused by the oversized molecules of conventional ADCs have demonstrated enhanced permeability and anticancer potential in solid tumors [76]. Some ADCs can also effectively address the problem of epigenetic heterogeneity by stimulating antitumor activity against cancer cells surrounding target antigen-expressing cells through a bystander effect. Several studies have shown that cleavable junctions and hydrophobic payloads are key to the realization of the bystander effect, which will be an important direction for the development of novel ADCs [77, 78].

### Linker optimization

Linkers are important components of ADCs, which play a key role in ADCs stability, efficacy, and safety by covalently linking monoclonal antibodies to cytotoxic payloads [79, 80]. Two main types of linkers exist: cleavable and non-cleavable linkers, while cleavable linkers include chemically unstable and enzyme-sensitive linkers. Among them, chemically unstable linkers can undergo cleavage in the acidic environment of tumors to release drugs efficiently, but their stability in the blood circulation is relatively poor, which usually leads to premature drug delivery [73, 81, 82]. The emergence of enzyme-sensitive linkers has solved this problem by improving stability and delivery efficiency through specific cleavage using specific enzymes overexpressed in tumors [83, 84]. Non-cleavable linkers release the drug by enzymatic complete degradation within the lysosome, which has the advantage of high stability and low off-target toxicity, but their low release efficiency and inability to produce a bystander effect need to be optimized by optimizing the payload structure to improve the efficacy [85].

### Payload optimization

Payloads improvement is a key component in the ADCs optimization process. Traditional payloads, such as microtubule inhibitors and DNA-targeting agents, have faced challenges due to their toxicity and dose limitations [76, 86]. In recent years, the successful application of topoisomerase I inhibitors as novel payloads has significantly enhanced the efficacy of ADCs including T-Dxd and SG, which achieve higher efficacy through a high drug-antibody ratio (DAR) [87–89]. Meanwhile, the introduction of low-toxicity molecules and improvements in linker technology have enhanced the stability and therapeutic window of ADCs [86]. In addition, to meet the need for tumor specificity and high potency, the selection of payloads usually favors molecules with efficient activity that can act at low concentrations, usually with IC50 values in the nanomolar to picomolar range [90].

The therapeutic regimen of ADCs in combination with ICIs has shown a remarkable trend in solid tumor treatment in recent years. This combination therapy has shown promising results in several solid tumor types (e.g., NSCLC, BC, gastric cancer, etc.), especially in refractory tumors and tumors with high immune tolerance, where the combination therapy has demonstrated enhanced anti-tumor activity. However, there are still some limitations that need to be addressed in order to achieve optimal results. Although preclinical and early clinical data suggest significant synergistic effects of this combination therapy, particularly in reducing tumor growth and enhancing immune response, the issue of drug resistance remains. Although some early clinical trials have demonstrated the potential of combination therapy with ADCs and ICIs in cancers such as HER-2-positive breast cancer and non-small-cell lung cancer, the inconsistency of effects between different tumor types, as well as the potential for serious adverse events, highlight the need for further optimization of treatment regimens. Future studies should focus on optimizing dosing regimens, selecting appropriate biomarkers, and exploring the safety of these combination therapies in a broader patient population to ensure more sustainable and effective treatment options.

## Conclusion

In summary, the combination of ADC and immunotherapy demonstrates significant synergistic potential, not only directly killing tumor cells through targeted delivery of cytotoxic drugs, but also inducing ICD and enhancing immune responses in the tumor microenvironment. Both preclinical and clinical studies have demonstrated that this synergistic effect helps to overcome tumor heterogeneity, reduce treatment resistance and improve patient prognosis [91]. Early clinical trials have shown that this combination strategy has demonstrated excellent anti-tumor activity and overall manageable toxicity in a variety of solid tumors. However, there is still a need to be vigilant about possible overlapping toxicity issues and to enhance monitoring of treatment safety and long-term outcomes. As relevant data are currently limited, support for optimizing clinical practice and improving patient care through continued pharmacovigilance and the accumulation of real-world evidence is still needed. In conclusion, the combination of ADCs and immunotherapy provides an exciting new direction for cancer treatment and is expected to become an important component of individualized therapy and drive overall progress in the treatment of refractory cancers (Figs. 1 and 2).

## Data Availability

All data generated or analyzed during this study are included in this published article. Data are available upon reasonable request. The data supporting this study’s findings are available from the corresponding author upon reasonable request.

## References

[1] Robert C, Ribas A, Hamid O, Daud A, Wolchok JD, Joshua AM, Hwu WJ, Weber JS, Gangadhar TC, Joseph RW, Dronca R. Durable complete response after discontinuation of pembrolizumab in patients with metastatic melanoma. J Clin Oncol. 2018;36(17):1668–74. 29283791 10.1200/JCO.2017.75.6270

[2] Jenkins RW, Barbie DA, Flaherty KT. Mechanisms of resistance to immune checkpoint inhibitors. Br J Cancer. 2018;118(1):9–16. 29319049 10.1038/bjc.2017.434 PMC5765236

[3] Sharma P, et al. Primary, adaptive, and acquired resistance to cancer immunotherapy. Cell. 2017;168(4):707–23. 28187290 10.1016/j.cell.2017.01.017 PMC5391692

[4] Chau CH, Steeg PS, Figg WD. Antibody–drug conjugates for cancer. Lancet. 2019;394(10200):793–804. 31478503 10.1016/S0140-6736(19)31774-X

[5] Drago JZ, Modi S, Chandarlapaty S. Unlocking the potential of antibody–drug conjugates for cancer therapy. Nat Rev Clin Oncol. 2021;18(6):327–44. 33558752 10.1038/s41571-021-00470-8 PMC8287784

[6] Pinto A, Guarini C, Giampaglia M, Sanna V, Melaccio A, Lanotte L, Santoro AN, Pini F, Cusmai A, Giuliani F, Gadaleta-Caldarola G. Synergizing immunotherapy and antibody–drug conjugates: new horizons in breast cancer therapy. Pharmaceutics. 2024;16(9):1146. 39339183 10.3390/pharmaceutics16091146 PMC11435286

[7] Verma S, et al. Trastuzumab emtansine for HER-2-positive advanced breast cancer. N Engl J Med. 2012;367(19):1783–91. 23020162 10.1056/NEJMoa1209124 PMC5125250

[8] Wei Q, Li P, Yang T, Zhu J, Sun L, Zhang Z, Wang L, Tian X, Chen J, Hu C, Xue J. The promise and challenges of combination therapies with antibody–drug conjugates in solid tumors. J Hematol Oncol. 2024;17(1):1. 38178200 10.1186/s13045-023-01509-2 PMC10768262

[9] Saini KS, et al. Antibody–drug conjugates, immune-checkpoint inhibitors, and their combination in breast cancer therapeutics. Expert Opin Biol Ther. 2021;21(7):945–62. 34043927 10.1080/14712598.2021.1936494

[10] Singh SK, Luisi DL, Pak RH. Antibody–drug conjugates: design, formulation and physicochemical stability. Pharm Res. 2015;32(11):3541–71. 25986175 10.1007/s11095-015-1704-4

[11] Mer AH, Mirzaei Y, Misamogooe F, Bagheri N, Bazyari A, Keshtkaran Z, Meyfour A, Shahedi A, Amirkhani Z, Jafari A, Barpour N. Progress of antibody–drug conjugates (ADCs) targeting c-Met in cancer therapy; insights from clinical and preclinical studies. Drug Deliv Trans Res. 2024;14(11):2963–88. 10.1007/s13346-024-01564-3 38597995

[12] Khosravanian MJ, Mirzaei Y, Mer AH, Keyhani-Khankahdani M, Abdinia FS, Misamogooe F, Amirkhani Z, Bagheri N, Meyfour A, Jahandideh S, Barpour N. Nectin-4-directed antibody–drug conjugates (ADCs): spotlight on preclinical and clinical evidence. Life Sci. 2024;352:122910. 39002610 10.1016/j.lfs.2024.122910

[13] Beck A, et al. Strategies and challenges for the next generation of antibody–drug conjugates. Nat Rev Drug Discov. 2017;16(5):315–37. 28303026 10.1038/nrd.2016.268

[14] Nicolò E, Giugliano F, Ascione L, Tarantino P, Corti C, Tolaney SM, Cristofanilli M, Curigliano G. Combining antibody–drug conjugates with immunotherapy in solid tumors: current landscape and future perspectives. Cancer Treatment Rev. 2022;106:102395. 10.1016/j.ctrv.2022.102395 35468539

[15] Garg AD, Galluzzi L, Apetoh L, Baert T, Birge RB, Bravo-San Pedro JM, Breckpot K, Brough D, Chaurio R, Cirone M, Coosemans A. Molecular and translational classifications of DAMPs in immunogenic cell death. Front Immunol. 2015;6:588. 26635802 10.3389/fimmu.2015.00588 PMC4653610

[16] Galluzzi L, et al. Immunogenic cell death in cancer and infectious disease. Nat Rev Immunol. 2017;17(2):97–111. 27748397 10.1038/nri.2016.107

[17] Li L, Ai L, Jia L, Zhang L, Lei B, Zhang Q. High score of LDH plus dNLR predicts poor survival in patients with HER2-positive advanced breast cancer treated with trastuzumab emtansine. BMC Cancer. 2022;22:1–3. 34980025 10.1186/s12885-021-09131-6 PMC8722106

[18] Waks AG, Keenan TE, Li T, Tayob N, Wulf GM, Richardson ET III, Attaya V, Anderson L, Mittendorf EA, Overmoyer B, Winer EP. Phase Ib study of pembrolizumab in combination with trastuzumab emtansine for metastatic HER2-positive breast cancer. Jfor Immunother Cancer. 2022;10(10):e005119. 10.1136/jitc-2022-005119 PMC9577940 36252998

[19] Martin K, et al. The microtubule-depolymerizing agent ansamitocin P3 programs dendritic cells toward enhanced anti-tumor immunity. Cancer Immunol Immunother. 2014;63(9):925–38. 24906866 10.1007/s00262-014-1565-4 PMC11029065

[20] Torres ETR, Emens LA. Emerging combination immunotherapy strategies for breast cancer: dual immune checkpoint modulation, antibody–drug conjugates and bispecific antibodies. Breast Cancer Res Treat. 2022;191(2):291–302. 34716871 10.1007/s10549-021-06423-0

[21] Müller P, Kreuzaler M, Khan T, Thommen DS, Martin K, Glatz K, Savic S, Harbeck N, Nitz U, Gluz O, von Bergwelt-Baildon M. Trastuzumab emtansine (T-DM1) renders HER2+ breast cancer highly susceptible to CTLA-4/PD-1 blockade. Sci Trans Med. 2015;7(315):315ra188. 10.1126/scitranslmed.aac4925 26606967

[22] Müller P, et al. Microtubule-depolymerizing agents used in antibody–drug conjugates induce antitumor immunity by stimulation of dendritic cells. Cancer Immunol Res. 2014;2(8):741–55. 24916470 10.1158/2326-6066.CIR-13-0198

[23] Kitai Y, et al. DNA-containing exosomes derived from cancer cells treated with topotecan activate a STING- dependent pathway and reinforce antitumor immunity. J Immunol. 2017;198(4):1649–59. 28069806 10.4049/jimmunol.1601694

[24] Tanaka H, et al. Classification of chemotherapeutic agents based on their differentialin vitroeffects on dendritic cells. Can Res. 2009;69(17):6978–86. 10.1158/0008-5472.CAN-09-1101 PMC2769260 19706756

[25] Iwata TN, et al. A HER-2-targeting antibody–drug conjugate, trastuzumab deruxtecan (DS-8201a), enhances antitumor immunity in a mouse model. Mol Cancer Ther. 2018;17(7):1494–503. 29703841 10.1158/1535-7163.MCT-17-0749

[26] McKenzie JA, Mbofung RM, Malu S, Zhang M, Ashkin E, Devi S, Williams L, Tieu T, Peng W, Pradeep S, Xu C. The Effect of Topoisomerase I Inhibitors on the Efficacy of T-Cell-Based Cancer Immunotherapy. JNCI: J Nat Cancer Inst. 2018;110(7):777–86. 29267866 10.1093/jnci/djx257 PMC6037061

[27] D’Amico L, Menzel U, Prummer M, Müller P, Buchi M, Kashyap A, Haessler U, Yermanos A, Gébleux R, Briendl M, Hell T. A novel anti-HER2 anthracycline-based antibody–drug conjugate induces adaptive anti-tumor immunity and potentiates PD-1 blockade in breast cancer. J Immunother Cancer. 2019;7:1–5. 30665463 10.1186/s40425-018-0464-1 PMC6341578

[28] Huang L, Wang R, Xie K, Zhang J, Tao F, Pi C, Feng Y, Gu H, Fang J. A HER2 target antibody drug conjugate combined with anti-PD-(L) 1 treatment eliminates hHER2+ tumors in hPD-1 transgenic mouse model and contributes immune memory formation. Breast cancer research and treatment. 2022;1-1. 10.1007/s10549-021-06384-4 34657203

[29] Iyer G, Al-Ahmadie H, Schultz N, Hanrahan AJ, Ostrovnaya I, Balar AV, Kim PH, Lin O, Weinhold N, Sander C, Zabor EC. Prevalence and Co-occurrence of actionable genomic alterations in high-grade bladder cancer. J Clin Oncol. 2013;31(25):3133–40. 23897969 10.1200/JCO.2012.46.5740 PMC3753703

[30] Strickler JH, et al. Diagnosis and treatment of ERBB2-positive metastatic colorectal cancer a review. JAMA Oncol. 2022;8(5):760–9. 35238866 10.1001/jamaoncol.2021.8196

[31] Li BT, et al. HER-2 Amplification and HER-2 mutation are distinct molecular targets in lung cancers. J Thorac Oncol. 2016;11(3):414–9. 26723242 10.1016/j.jtho.2015.10.025 PMC4698879

[32] Arkhipov A, Shan Y, Kim ET, Dror RO, Shaw DE. Her2 activation mechanism reflects evolutionary preservation of asymmetric ectodomain dimers in the human EGFR family. Elife. 2013;2:e00708. 23878723 10.7554/eLife.00708 PMC3713454

[33] Moasser MM. The oncogene HER-2: its signaling and transforming functions and its role in human cancer pathogenesis. Oncogene. 2007;26(45):6469–87. 17471238 10.1038/sj.onc.1210477 PMC3021475

[34] Yu J, Li M, Liu X, Wu S, Li R, Jiang Y, Zheng J, Li Z, Xin K, Xu Z, Li S. Implementation of antibody–drug conjugates in HER2-positive solid cancers: recent advances and future directions. Biomed Pharmacother. 2024;174:116522. 38565055 10.1016/j.biopha.2024.116522

[35] Emens LA, et al. Trastuzumab emtansine plus Atezolizumab versus trastuzumab emtansine plus placebo in previously treated, HER-2-positive advanced breast cancer (KATE2): a phase 2, multicentre, randomized, double-blind trial. Lancet Oncology. 2020;21(10):1283–95. 33002436 10.1016/S1470-2045(20)30465-4

[36] Loi S, et al. Pembrolizumab plus trastuzumab in trastuzumab-resistant, advanced, HER-2-positive breast cancer (PANACEA): a single-arm, multicentre, phase 1b–2 trial. Lancet Oncol. 2019;20(3):371–82. 30765258 10.1016/S1470-2045(18)30812-X

[37] SC et al. A Phase Ib trial of durvalumab in combination with trastuzumab in HER-2-positive. 10.1634/theoncologist.2019-0321 PMC6853090 31420468

[38] Andre F, et al. Trastuzumab deruxtecan versus treatment of physician’s choice in patients with HER-2-positive metastatic breast cancer (DESTINY- Breast02): a randomized, open-label, multicentre, 3 trial. Lancet. 2023;401(10390):1773–85. 37086745 10.1016/S0140-6736(23)00725-0

[39] Cortes J, et al. Trastuzumab deruxtecan versus trastuzumab emtansine for breast cancer. N Engl J Med. 2022;386(12):1143–54. 35320644 10.1056/NEJMoa2115022

[40] Hamilton E, Galsky MD, Ochsenreither S, Del Conte G, Martín M, De Miguel MJ, Yu EY, Williams A, Gion M, Tan AR, Agrawal L. Trastuzumab deruxtecan with nivolumab in HER2-expressing metastatic breast or urothelial cancer: analysis of the phase Ib DS8201-A-U105 study. Clin Cancer Res. 2024;30(24):5548–58. 39405343 10.1158/1078-0432.CCR-24-1513 PMC11647201

[41] Meric-Bernstam F, Makker V, Oaknin A, Oh DY, Banerjee S, González-Martín A, Jung KH, Ługowska I, Manso L, Manzano A, Melichar B. Efficacy and safety of trastuzumab deruxtecan in patients with HER2-expressing solid tumors: primary results from the DESTINY-PanTumor02 phase II trial. J Clin Oncol. 2024;42(1):47–58. 37870536 10.1200/JCO.23.02005 PMC10730032

[42] Modi S, et al. Trastuzumab deruxtecan in previously treated HER-2-positive breast cancer. N Engl J Med. 2020;382(7):610–21. 31825192 10.1056/NEJMoa1914510 PMC7458671

[43] Cheema P, Hartl S, Koczywas M, Hochmair M, Shepherd FA, Chu Q, Galletti G, Gustavson M, Iyer S, Barrett JC, Evans B. 695 Efficacy and safety of trastuzumab deruxtecan (T-DXd) with durvalumab in patients with non-small cell lung cancer (HER2 altered NSCLC) who progressed on anti-PD1/PD-L1 therapy (HUDSON).

[44] Italiano A et al. 2024;** 24**

[45] Andre F, Hamilton EP, Loi S, Anders CK, Schmid P, Stroyakovskiy D, Villanueva R, Pedrini JL, Doval DC, Zurawski B, Chen SC. DESTINY-Breast07: Dose-expansion interim analysis of T-DXd monotherapy and T-DXd+ pertuzumab in patients with previously untreated HER2+ mBC.

[46] Janjigian YY et al. 2024;**35**

[47] Peng Z, et al. Efficacy and safety of a novel anti-HER-2 therapeutic antibody RC48 in patients with HER-2-overexpressing, locally advanced or metastatic gastric or gastroesophageal junction cancer: a single-arm phase II study. Cancer Commun. 2021;41(11):1173–82. 10.1002/cac2.12214 PMC8626607 34665942

[48] Wang Y, Gong J, Wang A, Wei J, Peng Z, Wang X, Zhou J, Qi C, Liu D, Li J, Lu M. Disitamab vedotin (RC48) plus toripalimab for HER2-expressing advanced gastric or gastroesophageal junction and other solid tumours: a multicentre, open label, dose escalation and expansion phase 1 trial. EClinicalMedicine. 2024;68. 10.1016/j.eclinm.2023.102415 PMC10789637 38235421

[49] Zhou L, Yang KW, Zhang S, Yan XQ, Li SM, Xu HY, Li J, Liu YQ, Tang BX, Chi ZH, Si L. Disitamab vedotin plus toripalimab in patients with locally advanced or metastatic urothelial carcinoma (RC48-C014): a phase Ib/II dose-escalation and dose-expansion study. Ann Oncol. 2025;36(3):331–9. 39662628 10.1016/j.annonc.2024.12.002

[50] Liu Y, et al. Trop-2-targeted molecular imaging in solid tumors: current advances and future outlook. Mol Pharm. 2024;21(12):5909–28. 39537365 10.1021/acs.molpharmaceut.4c00848 PMC11832138

[51] Zhou L, Yang K, Zhang S, Yan X, Li S, Xu H, Li J, Chi Z, Mao L, Lian B, Bixia T. 1979P Disitamab vedotin (DV) plus toripalimab (T) in unresectable locally advanced or metastatic urothelial carcinoma (la/mUC): Long-term outcomes from a phase Ib/II study. Ann Oncol. 2024;35:S1145.

[52] Garrido-Castro AC et al. SACI-IO HR plus: a randomized phase II trial of sacituzumab govitecan with or without Pembrolizumab in patients with metastatic hormone receptor-positive/HER-2-negative breast cancer. Journal of Clinical Oncology, 2024;**42** (17_SUPPL): p. LBA1004-LBA1004. 10.1016/j.annonc.2026.06.012 42323161

[53] Schmid P, Loi S, De la Cruz Merino L, Yerushalmi R, Im SA, Sonnenblick A, Garcia MM, Candilejo IM, Kennedy LC, Griffiths KL, Schwab R. 181O Interim analysis (IA) of the atezolizumab (atezo)+ sacituzumab govitecan (SG) arm in patients (pts) with triple-negative breast cancer (TNBC) in MORPHEUS-pan BC: A phase Ib/II study of multiple treatment (tx) combinations in pts with locally advanced/metastatic BC (LA/mBC). ESMO Open. 2024;9.

[54] Cappuzzo F, Patel J, Cho BC, Dols MC, Cabanillas RR, Baz DV, Pradera JF, Neal J, Garon EB, Mekan S, Safavi F. 60P Sacituzumab govitecan (SG)+ pembrolizumab (pembro) in first-line (1L) metastatic non-small cell lung cancer (mNSCLC): Efficacy results by histology from the EVOKE-02 study. ESMO Open. 2024;9.

[55] Schmid P, et al. Datopotamab deruxtecan (Dato-DXd) plus durvalumab (D) as first-line (1L) treatment for unresectable locally advanced/metastatic triple-negative breast cancer (a/mTNBC): updated results from BEGONIA, a phase Ib/II study. Ann Oncol. 2023;34:S337–S337. 10.1016/j.annonc.2026.05.693 42167437

[56] Levy BP, Paz-Ares LG, Su WC, Herbert SM, Yang TY, Tolcher AW, Lou Y, Zenke Y, Cortinovis DL, Felip E, Domine Sr M. Datopotamab deruxtecan (Dato-DXd) plus pembrolizumab (pembro) with or without platinum chemotherapy (Pt-CT) as first-line (1L) therapy for advanced non-small cell lung cancer (aNSCLC): Subgroup analysis from TROPION-Lung02. 10.1016/j.jtho.2026.103688 41871716

[57] Fang W, Wang Q, Cheng Y, Luo Y, Qu X, Zhu H, Ding Z, Li X, Wu L, Wang Y, Hu S. Sacituzumab tirumotecan (SKB264/MK-2870) in combination with KL-A167 (anti-PD-L1) as first-line treatment for patients with advanced NSCLC from the phase II OptiTROP-Lung01 study.

[58] Powles T, et al. Enfortumab vedotin and pembrolizumab in untreated advanced urothelial cancer. N Engl J Med. 2024;390(10):875–88. 38446675 10.1056/NEJMoa2312117

[59] Swiecicki PL, Yilmaz E, Rosenberg AJ, Fujisawa T, Bruce JY, Meng C, Wozniak M, Zhao Y, Mihm M, Kaplan J, Gorla S. Phase II trial of enfortumab vedotin in patients with previously treated advanced head and neck cancer. J Clin Oncol. 2025;43(5):578–88. 39481054 10.1200/JCO.24.00646 PMC11809727

[60] Porter RL, Veneris JT, Tayob N, West G, Polak M, Gardner J, Campos SM, Krasner CN, Lee EK, Liu JF, Stover E. A phase 2, two-stage study of mirvetuximab soravtansine (IMGN853) in combination with pembrolizumab in patients with microsatellite stable (MSS) endometrial cancer (EC).

[61] Vergote I, Van Nieuwenhuysen E, O’Cearbhaill RE, Westermann A, Lorusso D, Ghamande S, Collins DC, Banerjee S, Mathews CA, Gennigens C, Cibula D. Tisotumab vedotin in combination with carboplatin, pembrolizumab, or bevacizumab in recurrent or metastatic cervical cancer: results from the innovaTV 205/GOG-3024/ENGOT-cx8 study. J Clin Oncol. 2023;41(36):5536–49. 37651655 10.1200/JCO.23.00720 PMC10730069

[62] Abreu DR, Veillon R, Ravoire M, Gonzalez-Larriba JL, Orlandi FJ, Nagy T, Paz-Ares LG, Huang CH, Roubec J, Isambert N, Campelo MG. 1311P Phase II, open-label study of frontline tusamitamab ravtansine with pembrolizumab±chemotherapy in advanced non-squamous non-small cell lung cancer: updated results from CARMEN-LC05 trial. Ann Oncol. 2024;35:S834.

[63] Fan J, Zhou J, Shi G, Huang X, Gao Q, Liang F, Ren N, Shi Y, Yi Y, Wang Z, Yu Y. Two stage, multi-center trial of cadonilimab and LM-302 for patients with CLDN18. 2+ biliary tract cancer (BTC) that failed chemotherapy and PD-(L) 1 antibody (ZSAB-Calm).

[64] Hoimes CJ, Flaig TW, Milowsky MI, Friedlander TW, Bilen MA, Gupta S, Srinivas S, Merchan JR, McKay RR, Petrylak DP, Sasse C. Enfortumab Vedotin Plus Pembrolizumab in previously untreated advanced urothelial cancer. J Clin Oncol. 2023;41(1):22–31. 36041086 10.1200/JCO.22.01643 PMC10476837

[65] Camus P, Kudoh S, Ebina M. Interstitial lung disease associated with drug therapy. Br J Cancer. 2004;91:S18–23. 15340374 10.1038/sj.bjc.6602063 PMC2750811

[66] Conte P, Ascierto PA, Patelli G, Danesi R, Vanzulli A, Sandomenico F, Tarsia P, Cattelan A, Comes A, De Laurentiis M, Falcone A. Drug-induced interstitial lung disease during cancer therapies: expert opinion on diagnosis and treatment. ESMO Open. 2022;7(2):100404. 35219244 10.1016/j.esmoop.2022.100404 PMC8881716

[67] Janne PA, et al. Efficacy and safety of patritumab deruxtecan (HER3-DXd) in EGFR inhibitor-resistant, EGFR-mutated non- small cell lung cancer. Cancer Discov. 2022;12(1):74–89. 34548309 10.1158/2159-8290.CD-21-0715 PMC9401524

[68] Skeoch S, Weatherley N, Swift AJ, Oldroyd A, Johns C, Hayton C, Giollo A, Wild JM, Waterton JC, Buch M, Linton K. Drug-induced interstitial lung disease: a systematic review. J Clin Med. 2018;7(10):356. 30326612 10.3390/jcm7100356 PMC6209877

[69] Spira A, et al. Datopotamab deruxtecan (Dato-DXd; DS-1062), a TROP-2 ADC, in patients with advanced NSCLC: updated results of TROPION-PanTumor01 Phase 1 study. J Thorac Oncol. 2021;16(3):S106–7.

[70] Rugo HS, Tokunaga E, Iwata H, Petry V, Smit EF, Goto Y, Kim DW, Shitara K, Gruden JF, Modi S, Cortés J. 267MO Pooled analysis of trastuzumab deruxtecan (T-DXd) retreatment (RTx) after recovery from grade (Gr) 1 interstitial lung disease/pneumonitis (ILD). ESMO Open. 2024; 9. 10.1016/j.annonc.2025.07.015 40769277

[71] Nam S, Lim SM, Cho BC, Lee JB. Successful rechallenge of trastuzumab deruxtecan after drug-induced interstitial lung disease in a NSCLC with HER2 mutation: a case report. JTO Clin Res Rep. 2024;5(2):100628. 38298273 10.1016/j.jtocrr.2023.100628 PMC10825559

[72] Dumontet C, et al. Antibody–drug conjugates come of age in oncology. Nat Rev Drug Discovery. 2023;22(8):641–61. 37308581 10.1038/s41573-023-00709-2

[73] Tsuchikama K, et al. Exploring the next generation of antibody–drug conjugates. Nat Rev Clin Oncol. 2024;21(3):203–23. 38191923 10.1038/s41571-023-00850-2

[74] Gutierrez C, Schiff R. HER-2 biology, detection, and clinical implications. Arch Pathol Lab Med. 2011;135(1):55–62. 21204711 10.1043/2010-0454-RAR.1 PMC3242418

[75] Stepan LP, et al. Expression of Trop-2 cell surface glycoprotein in normal and tumor tissues: potential implications as a cancer therapeutic target. J Histochem Cytochem. 2011;59(7):701–10. 21551320 10.1369/0022155411410430 PMC3201164

[76] Grairi M, Le Borgne M. Antibody–drug conjugates: prospects for the next generation. Drug Discovery Today. 2024;29:104241. 39542204 10.1016/j.drudis.2024.104241

[77] Giugliano F, et al. Bystander effect of antibody–drug conjugates: fact or fiction? Curr Oncol Rep. 2022;24(7):809–17. 35305211 10.1007/s11912-022-01266-4

[78] Hosonaga M, Arima Y, Sampetrean O, Komura D, Koya I, Sasaki T, Sato E, Okano H, Kudoh J, Ishikawa S, Saya H. HER2 heterogeneity is associated with poor survival in HER2-positive breast cancer. Int J Mol Sci. 2018;19(8):2158. 30042341 10.3390/ijms19082158 PMC6121890

[79] Chudasama V, Maruani A, Caddick S. Recent advances in the construction of antibody–drug conjugates (vol 8, pg 114, 2016). Nat Chem. 2016;8(3):283–283. 26791893 10.1038/nchem.2415

[80] Perez HL, et al. Antibody–drug conjugates: current status and future directions. Drug Discovery Today. 2014;19(7):869–81. 24239727 10.1016/j.drudis.2013.11.004

[81] Kostova V, Désos P, Starck JB, Kotschy A. The chemistry behind ADCs. Pharmaceuticals. 2021;14(5):442. 34067144 10.3390/ph14050442 PMC8152005

[82] Senter PD. Potent antibody drug conjugates for cancer therapy. Curr Opin Chem Biol. 2009;13(3):235–44. 19414278 10.1016/j.cbpa.2009.03.023

[83] Bargh JD, et al. Cleavable linkers in antibody–drug conjugates. Chem Soc Rev. 2019;48(16):4361–74. 31294429 10.1039/c8cs00676h

[84] Gondi CS, Rao JS. Cathepsin B as a cancer target. Expert Opin Ther Targets. 2013;17(3):281–91. 23293836 10.1517/14728222.2013.740461 PMC3587140

[85] McCombs JR, Owen SC. Antibody drug conjugates: design and selection of linker. Payload Conjug Chem Aaps J. 2015;17(2):339–51. 10.1208/s12248-014-9710-8 PMC4365093 25604608

[86] Conilh L, Sadilkova L, Viricel W, Dumontet C. Payload diversification: a key step in the development of antibody–drug conjugates. J Hematol Oncol. 2023;16(1):3. 36650546 10.1186/s13045-022-01397-y PMC9847035

[87] Keam SJ. Trastuzumab deruxtecan: first approval. Drugs. 2020;80(5):501–8. 32144719 10.1007/s40265-020-01281-4

[88] Ogitani Y, et al. DS-8201a, A novel HER-2-targeting ADC with a novel DNA topoisomerase I inhibitor, demonstrates a promising antitumor efficacy with differentiation from T-DM1. Clin Cancer Res. 2016;22(20):5097–108. 27026201 10.1158/1078-0432.CCR-15-2822

[89] Syed YY. Sacituzumab govitecan: first approval. Drugs. 2020;80(10):1019–25. 32529410 10.1007/s40265-020-01337-5 PMC7288263

[90] Zhao P, et al. Recent advances of antibody drug conjugates for clinical applications. Acta Pharmaceutica Sinica B. 2020;10(9):1589–600. 33088681 10.1016/j.apsb.2020.04.012 PMC7564033

[91] Nucera S, Conti C, Martorana F, Wilson B, Genta S. Antibody–drug conjugates to promote immune surveillance: lessons learned from breast cancer. Biomedicines. 2024;12(7):1491. 39062065 10.3390/biomedicines12071491 PMC11274676

